# RIPK3 and caspase 8 collaborate to limit herpes simplex encephalitis

**DOI:** 10.1371/journal.ppat.1010857

**Published:** 2022-09-19

**Authors:** Hongyan Guo, Heather S. Koehler, Edward S. Mocarski, Richard D. Dix

**Affiliations:** 1 Viral Immunology Center, Department of Biology, Georgia State University, Atlanta, Georgia, United States of America; 2 Department of Microbiology and Immunology, Emory University School of Medicine, Atlanta, Georgia, United States of America; 3 Department of Ophthalmology, Emory University School of Medicine, Atlanta, Georgia, United States of America; University of North Carolina at Chapel Hill, UNITED STATES

## Abstract

Invasion of the brain by herpes simplex virus 1 (HSV1) can lead to the development of herpes simplex encephalitis (HSE) that is often associated with significant morbidity and mortality regardless of therapeutic intervention. Both virus and host immune factors dictate HSE onset and progression. Because programmed cell death pathways including necroptosis are important antiviral defense mechanisms in HSV1-associated peripheral diseases, they might also play critical roles in HSV1 neuropathogenesis. HSV1-encoded ICP6 prevents receptor-interacting protein kinase 3 (RIPK3)-mediated necroptosis during infection of human cells, but it also acts as a species-dependent inducer of necroptosis in murine cells and thereby restricts virus replication. We therefore used an established mouse model of HSE to investigate RIPK3-mediated necroptosis impact on HSV1 neuropathogenesis. Following corneal HSV1 inoculation, RIPK3 knockout mice showed increased susceptibility to HSE when compared with wildtype mice indicating RIPK3 helps to limit HSE progression. RIPK3-mediated defense against HSE was found to be independent of the kinase domain necessary to drive necroptosis implicating that a death independent function of RIPK3 protects against HSE. Conversely the pro-necroptotic kinase function RIPK3 served to limit viral replication in corneal tissue implicating a tissue-specific RIPK3 function in limiting HSV1. Further evaluation of the kinase-independent mechanism to restrict HSE revealed that the RIPK3 signaling partner, caspase 8, contributes to limiting HSE neuropathogenesis. Increased HSE susceptibility from loss of caspase 8 and RIPK3 correlated with decreased levels of chemokines, cytokines, and antiviral lymphocytes recruitment to the brain. We conclude that RIPK3 contributes toward host control of HSV1 replication in a tissue-specific fashion. Whereas RIPK3-mediated necroptosis restricts virus replication within the cornea, kinase-independent induction of inflammation by RIPK3 in collaboration with caspase 8 restricts virus replication within the brain during HSE neuropathogenesis.

## Author summary

Necroptosis is a programmed cell death pathway whose function is to restrict virus replication within tissue and thereby prevent or minimize clinical disease. RIPK3 is a kinase critical to the activation of necroptosis, but may contribute to the restriction of virus replication in other kinase-independent ways. We therefore performed studies to investigate possible mechanisms whereby RIPK3 might contribute to the neuropathogenesis of a fatal inflammation of the brain called herpes simplex encephalitis (HSE) caused by herpes simplex virus 1 (HSV1). Using a mouse model of HSE in which HSV1 infection is done by cornea inoculation with subsequent neural virus spread to the brain, we showed that RIPK3 restricts virus replication by different mechanisms within cornea and brain in a tissue-specific fashion. Whereas HSV1 replication is restricted within corneal tissue by RIPK3 kinase-mediated necroptosis, HSV1 replication is restricted within brain tissue by RIPK3 kinase-independent activity that requires collaboration with caspase 8. This collaboration stimulates the recruitment of antiviral immune responses within the brain that leads to necroptosis-independent inflammation which provides protection against HSV1-induced HSE. Our findings extend the role of RIPK3 during HSV1 neuropathogenesis and may open up new avenues for the treatment of HSE.

## Introduction

Herpes simplex virus 1 (HSV1) is a medically significant neurotropic pathogen that exhibits an affinity for components of the peripheral nervous system and, on occasion, invades the central nervous system (CNS) of immunologically normal adults to cause a severe acute necrotizing encephalitis called herpes simplex encephalitis (HSE) [1]. Without antiviral intervention, HSE is often fatal with survivors displaying moderate to severe neurologic sequelae [1, 2]. Antiviral treatment reduces virus replication in the brain and consequently reduces HSE mortality and morbidity [3] but, even after recovery, the viral genome can remain in the CNS for life [4].

The tissue necrosis associated with HSE is thought to arise from several pathogenic events in which both virus and host factors contribute. Virus-induced cytopathology due to HSV1 replication within the brain parenchyma contributes directly to the tissue destruction associated with HSE [5, 6]. The potential destructive nature of host antiviral immune mechanisms, including cellular immunity, also has been suggested by animal investigations [7] as well as clinical studies [8, 9]. On the other hand, evidence for host cell defense against HSV1 infection of CNS tissues has been provided by prior genetic studies of isolated patient samples showing the induction of the Toll-like receptor (TLR) 3-dependent pathway of interferon (IFN)-α/β and -λ [10–14]. Additional in vivo studies using knockout mouse models have contributed further to our understanding of these and other signaling pathways that restrict virus replication in the CNS [15–19]. Improved understanding of the pathogenesis of HSE is likely to lead to more effective therapies to manage this life-threatening disease.

A possible role for programmed cell death (PCD) pathways during the onset of HSE and subsequent development of neuropathology has remained largely unexplored. One such PCD pathway, necroptosis, contributes to innate immunity by eliminating virus-infected cells and restricting dissemination within the host [20–23]. Receptor interacting protein kinase 3 (RIPK3) mediates necroptosis, a caspase-independent inflammatory type of cell death that releases intracellular immunogenic contents [20, 24]. RIPK3 consists of a kinase domain at the amino terminus [20, 24, 25] and a RIP homotypic interaction motif (RHIM) at the carboxy terminus [26]. During initiation of necroptosis, RHIM-dependent activation of RIPK3 depends on one of three RHIM-containing adaptors: RIPK1 [20, 24, 25], TIR-domain-containing adapter-inducing interferon-β (TRIF) [27], or Z-nucleic acid binding protein 1 (ZBP1, also called DAI) [28]. Necroptosis is then executed following RIPK3 kinase-dependent phosphorylation of MLKL, a pseudokinase that dictates membrane disruption [29]. RIPK3 also has the capacity to trigger apoptosis via recruitment of RIPK1, Fas-associated via death domain (FADD), and caspase 8 [30] that may also result in activation of inflammatory cytokine transcription independently of RIPK3 kinase activity and cell death [31, 32]. This PCD-independent function was recently documented to be important during West Nile virus (WNV) infection of the CNS where RIPK3 enhanced protection by promoting neuronal expression of inflammatory chemokines [33]. Importantly, the contribution of pro-necrotic protein kinase activity of RIPK3 in HSE neuropathogenesis has not been addressed. It also remains unclear whether the observed inflammatory signaling patterns are virus-specific or tissue-specific.

Herein, we sought to determine the role of RIPK3-mediated innate signaling in HSE disease pathogenesis following corneal inoculation of the eye, a route that mimics one form of natural virus transmission [17]. We show for the first time that RIPK3-mediated necroptosis contributes to restricting HSV1 replication in corneal tissues, but, in the brain, a kinase-independent function of RIPK3 contributes to restricting HSV1 replication that is dependent on caspase 8. The resulting RIPK3 and caspase 8 collaboration drives the production of elevated chemokines and cytokines that are associated collectively with recruitment of antiviral leukocyte populations to the brain. Taken together, our findings implicate RIPK3 in host control over HSV1 infection through different mechanisms that act in tissue-specific ways.

## Results

### Mice deficient in RIPK3 show increased mortality following corneal HSV1 infection

**Fig 1:**
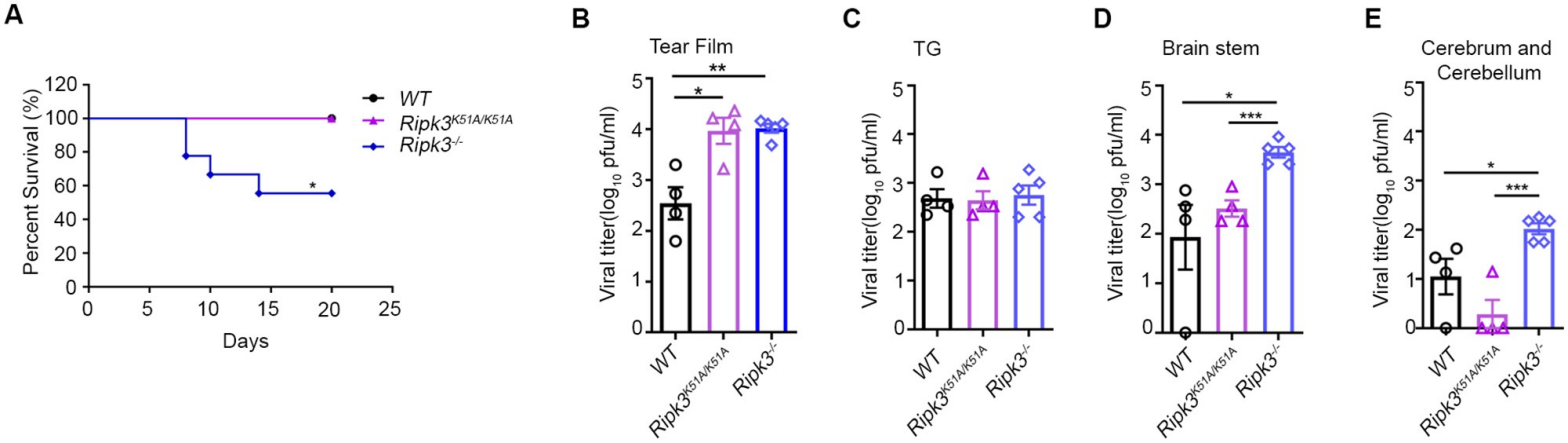
RIPK3 contributes to the pathogenesis of HSV-1 encephalitis, independent of its pro-necroptotic kinase activity. Corneas of 6-8-week-old WT, *Ripk3*^*−/−*^ and *Ripk3*^*K51A/K51A*^ mice were scarified and inoculated with 5 × 10^5^ pfu of HSV1 strain McKrae on the left eye. (A) Survival plot showing percent survival of WT (n = 7), *Ripk3*^*−/−*^ (n = 6), and *Ripk3*^*K51A/K51A*^(n = 9) mice after HSV1 infection. Tear film swab (B), trigeminal ganglion (TG) (C), brainstem (D), and (E) cerebrum and cerebellum tissues were collected at 5 dpi for determining viral titers (n = 4–5 per group). Values are expressed as means ± SEM (*P<0.05, **P<0.01, ***P<0.001).

We used throughout this investigation a well-established and clinically relevant mouse model of HSV1 neurologic infection. Prior work by others has shown that corneal inoculation of healthy mice with HSV1 results in virus replication within corneal tissue with subsequent neural spread of virus to the adjacent trigeminal ganglion and possible additional neural spread of virus into the brain parenchyma via the brainstem to cause HSE [34, 35]. To begin to assess the role of RIPK3 in controlling HSV1 neuropathogenesis, the corneal surface of eyes of *Ripk3*^*-/-*^ mice and wildtype (WT) C57BL/6J mice were inoculated with the McKrae strain of HSV1 and evaluated daily for 20 days for the development of death due to HSE. Whereas 100% of HSV1-infected WT mice survived, significant mortality was observed in HSV1-infected *Ripk3*^*-/-*^ mice with only 50% survival (Fig 1A).

### RIPK3 limits HSV1 pathogenesis in brain independently of pro-necroptotic kinase activity

RIPK3 is a well-recognized necroptosis mediator conferred by its kinase activity as well as acting as a scaffold to trigger either apoptosis or death-independent inflammation [30, 32]. To determine whether RIPK3 pro-necroptotic kinase activity contributes to HSV1 neuropathogenesis and control of HSE, *Ripk3*^*K51A/K51A*^ mice that produce RIPK3 but with inactive kinase activity were employed. Following infection with HSV1 by corneal inoculation, 100% of *Ripk3*^*K51A/K51A*^ mice survived, mirroring the survival of HSV1-infected WT mice but unlike HSV1-infected *Ripk3*^*-/-*^ mice that showed increased HSE susceptibility (Fig 1A).

**Fig 2:**
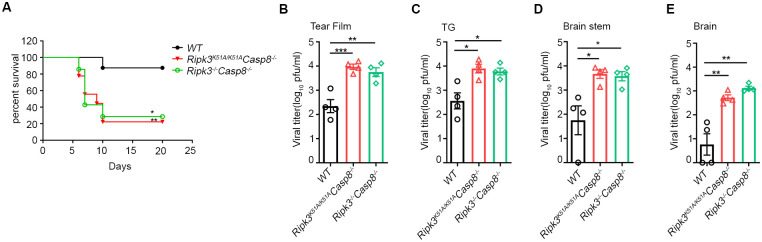
Caspase 8 contributes to RIPK3 protection within CNS against HSV1. Mice were infected as above described. (A) Plot showing percent survival of WT (n = 8), *Ripk3*^*-/-*^*Casp8*^*-/-*^ (n = 9) and *Ripk3*^*K51A/K51A*^*Casp8* ^*-/-*^ (n = 7) mice after HSV1 infection. Viral titers at 5 dpi in (B) tear film, (C) trigeminal ganglion (TG), (D) brainstem, and (E) combined cerebrum and cerebellum tissues were tested (n = 4–5 per group). Values are expressed as means ± SEM (*P<0.05, **P<0.01, ***P<0.001).

To further understand the contributions of RIPK3 toward HSV1 virus spread from cornea to brain during neuropathogenesis, infectious virus quantification studies were performed using HSV1-infected *Ripk3*^*K51A/K51A*^ mice, HSV1-infected *Ripk3*^*-/-*^ mice, and HSV1-infected WT mice. Quantification of infectious virus within tear films at 5 days after corneal inoculation as a measure of corneal HSV-1 replication [36] revealed significantly higher amounts of infectious virus produced from corneal tissues of HSV1-infected *Ripk3*^*K51A/K51A*^ mice and HSV1-infected *Ripk3*^*-/-*^ mice when compared with tear films collected from HSV1-infected WT mice (Fig 1B). These findings suggest that RIPK3-mediated necroptosis controls HSV1 infection within corneal tissues, the primary site of infection. Although equivalent amounts of infectious virus were found in trigeminal ganglia collected at 5 days after HSV1 infection of all three animal groups (Fig 2C), this was not the case for brainstem and whole brain consisting of combined cerebrum and cerebellum. Levels of virus replication within these CNS tissues collected from HSV1-infected *Ripk3*^*K51A/K51A*^ mice were significantly lower than those collected from HSV1-infected *Ripk3*^*-/-*^ mice but similar to those collected from HSV1-infected WT mice (Fig 2D and 2E) indicating that the kinase-specific activity of RIPK3 did not contribute to the restriction of HSV1 neuroinvasion and ultimate replication within the brain. Taken together, these results reveal a role for pro-necroptotic RIPK3 kinase activity in corneal tissue comprised of corneal epithelial and stromal cells, a finding consistent with observations of HSV1 infection in cultured mouse fibroblast and endothelial cells [37]. In sharp contrast, however, RIPK3 kinase activity appears to be dispensable for restricting virus replication within brain tissues, thereby implicating the RHIM-mediated scaffold function of RIPK3 in an antiviral host defense role against HSV1 infection as observed with other diseases [38].

### RIPK3-mediated restriction of HSV1 neuropathogenesis is dependent upon caspase 8

Because the scaffolding function of RIPK3 is known to recruit RIPK1-FADD-caspase 8 to mediate either apoptosis or inflammation [30, 32], we next sought to evaluate whether RIPK3 kinase independent protection from HSE is dependent on caspase 8. Caspase 8 deficient mice (*Casp8*^*-/-*^ mice) die during midgestation due to uncontrolled activation of RIPK3 but can be rescued by crossing to RIPK3 deficient mice yielding *Ripk3*^*-/-*^*Casp8*^*-/-*^mice or by disruption of the RIPK3 kinase activity yielding *Ripk3*^*K51A/K51A*^*Casp8*^*-/-*^ mice [39]. These double deficient mice were therefore evaluated for their susceptibility to HSV1 neuropathogenesis following corneal inoculation. In agreement with previous findings (Fig 1A), HSV1-infected WT mice were relatively resistant to death; 90% of the animals survived by 20 days postinfection (Fig 2A). In comparison, however, significantly enhanced mortality was observed in HSV1-infected *Ripk3*^*K51A/K51A*^ *Casp8*^*-/-*^ mice as well as HSV1-infected *Ripk3*^*-/-*^*Casp8*^*-/-*^mice with both animal groups showing only 20–30% survival by 20 days postinfection (Fig 2A). It is noteworthy that the level of mortality observed in HSV1-infected mice deficient in both RIPK3 and caspase 8 activity was substantially lower that than observed in HSV1-infected mice deficient in RIPK3 alone.

When infectious virus amounts were determined within tear films, trigeminal ganglia, brainstem and whole brain collected from all three animal groups at 5 days after corneal inoculation, HSV1-infected *Ripk3*^*K51A/K51A*^ *Casp8*^*-/-*^ mice and HSV1-infected *Ripk3*^*-/-*^*Casp8*^*-/-*^mice consistently showed significantly higher amounts of infectious virus for tears films and all neurologic tissues examined when compared with those examined for HSV1-infected WT mice (Fig 2B–2E). These results collectively implicate a role for caspase 8 during RIPK3-dependent restriction of HSV1 neuropathogenesis through its scaffold function.

### RIPK3 in collaboration with caspase 8 contribute to suppression of HSE neuropathology

**Fig 3:**
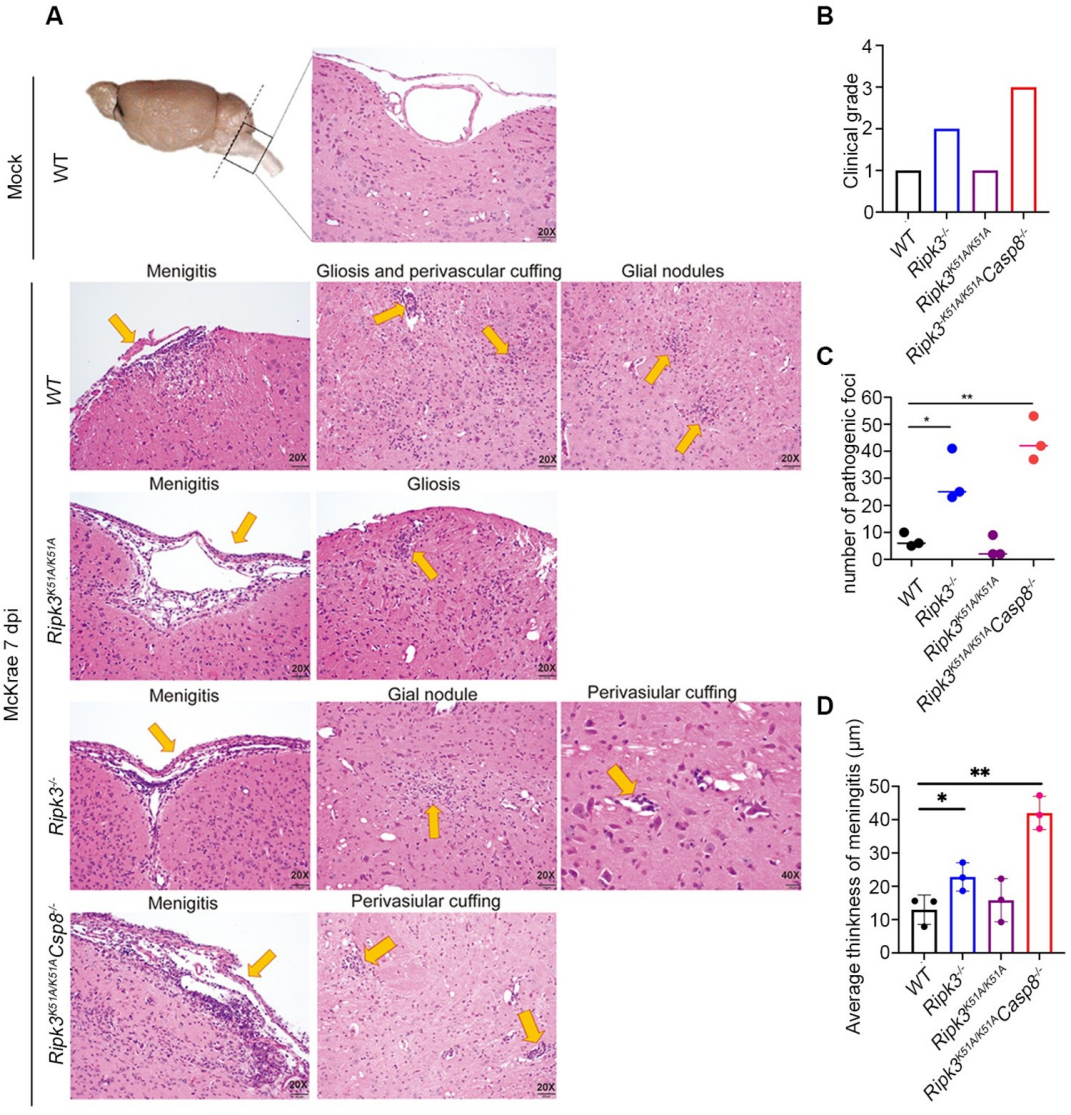
H&E sections of brainstem at 7 dpi. (A) Images represent examples of pathologic findings indicated on each panel. (B) Pathological scores of sections. (C) Number of glial nodules or perivascular cuffing within the parenchymal of the brainstems. (D) Measurement of the average thickness of the meningeal tissue at the ventral surface of the brainstem. Each dot represents different section. Distribution of meningeal thickness in micrometers. Values are expressed as means ± SEM (*P<0.05, **P<0.01, ***P<0.001).

Neuroinvasion of the mouse brain following corneal HSV1 inoculation occurs via the brainstem [34, 35]. We therefore evaluated how RIPK3 and caspase 8 might contribute to the onset of HSE by performing a comprehensive and quantitative histopathologic analysis of brainstem tissues collected at 7 days after corneal inoculation from HSE-susceptible HSV1-infected *Ripk3*^*-/-*^ mice and *Ripk3*^*K51A/K51A*^*Casp8*^*-/-*^ mice and compared with HSE-resistant HSV1-infected *Ripk3*^*K51A/K51A*^ mice and HSV1-infected WT mice. Entire brainstem sections from mock-infected WT mice served as controls. Histopathologic sections from each animal group were then evaluated by a pathologist in a blinded fashion and scored for degrees of neuropathology (0 for none detected, 1 for minimal pathology, 2 for mild pathology, 3 for moderate pathology, and 4 for severe pathology) using several HSE-associated pathologic markers. These markers included the appearance of microglial nodules (gliosis), perivascular cuffing, and meningeal inflammation. Results are summarized in Fig 3. In agreement with amounts of virus detected in the brainstems of these animal groups (Figs 1D and 2D), brainstem sections from HSV1-infected *Ripk3*^*-/-*^ and HSV1-infected *Ripk3*^*K51A/K51A*^*Casp8*^*-/-*^mice exhibited moderate to severe disease (Fig 3B) characterized by significant gliosis, moderate perivascular cuffing, and prominent meningeal mononuclear cell inflammation (Fig 3C and 3D). In contrast, brainstem sections from HSV1-infected *Ripk3*^*K51A/K51A*^ mice and HSV1-infected WT mice exhibited minimal disease pathology with only a few foci of gliosis, minimal perivascular cuffing, and relatively mild meningeal mononuclear cell inflammation. Thus, these histopathologic findings coupled with findings from parallel survival and virus quantification studies (Figs 1 and 2) support the conclusion that RIPK3 scaffold function through collaboration with caspase 8 together contribute to suppression of HSE-associated inflammation and subsequent neuropathology.

### RIPK3 regulates HSV1-induced chemokine and cytokine expression within brain tissues

**Fig 4:**
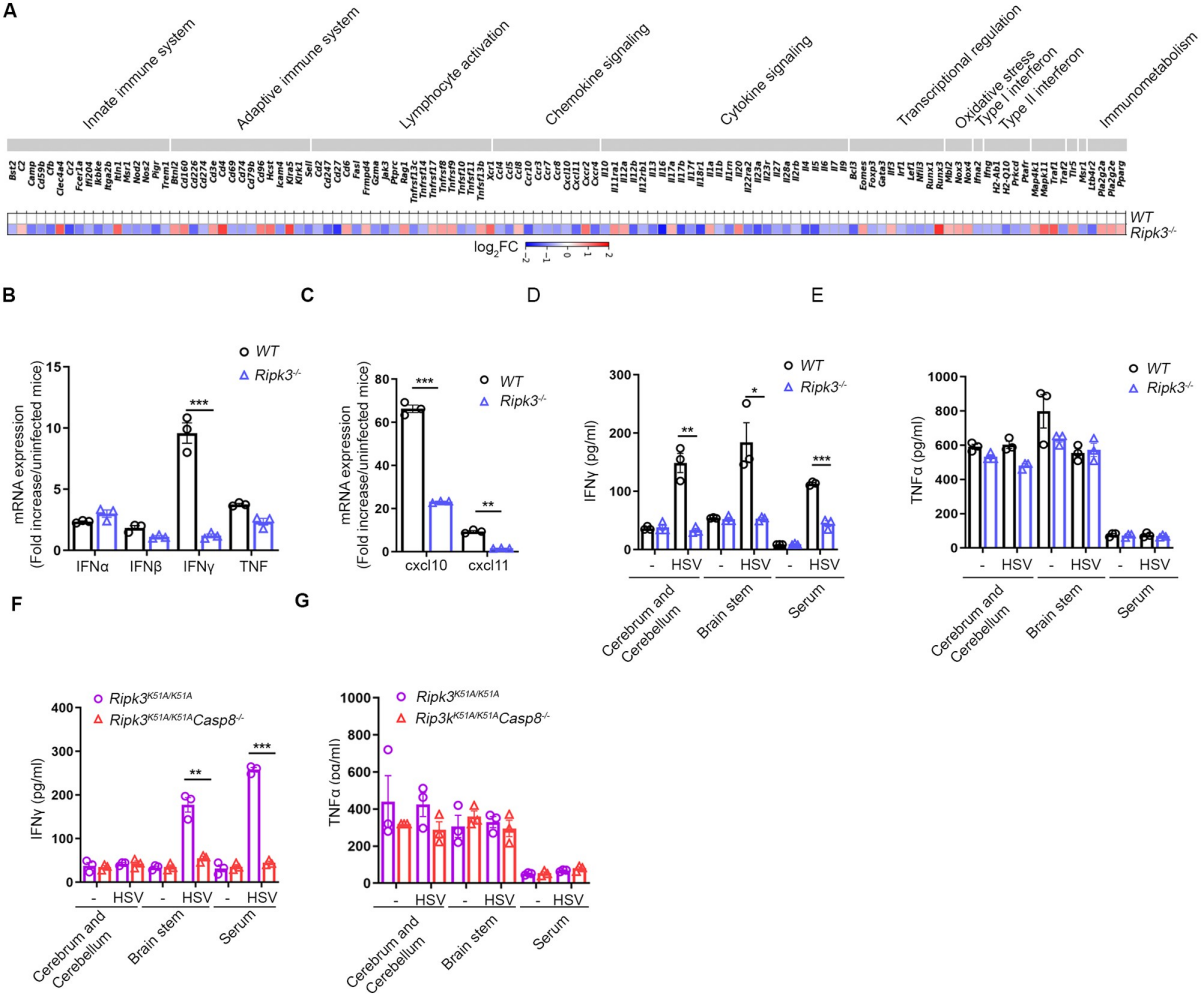
Impact of RIPK3 on tissue cytokine levels. (A) Heat map comparing mRNA patterns of immune-related genes in brain from WT and *Ripk3*^*−/−*^ mice (n = 3 for each group) at 5 dpi, where mean fold-change of each transcript was compared to normalized expression of all WT immune-related transcripts. Blue, white and red indicate down-regulated, unchanged and up-regulated expression, respectively. (B and C) mRNA levels of IFNs, cxcl10, cxcl11 were measured by qRT-PCR in brain homogenates of WT and *Ripk3*^*-/-*^ mice. (D and E) Levels of IFNγ and TNF were measured by ELISA in combined cerebrum and cerebellum or brainstem homogenates as well as serum of WT and *Ripk3*^*-/-*^ mice. (F and G) Levels of IFNγ and TNF were measured by ELISA in cerebrum and cerebellum, brainstem homogenates as well as serum of *Ripk3*^*K51A/K51A*^ and *Ripk3*^*K51A/K51A*^*Casp8* ^*-/-*^mice (n = 3 per group). *P < 0.05; **P < 0.01; ***P < 0.001.

A complex network of chemokines and cytokines produced in response to neurotropic virus infection shape the neuroimmune responses and consequent antiviral host defense within the CNS [33]. To obtain a more comprehensive view of the role RIPK3 signaling plays in controlling neuroinflammation, NanoString nCounter analysis was used to quantify and compare transcript levels for 564 immune-related genes in whole brains collected from HSV1-infected *Ripk3*^*-/-*^ mice and HSV1-infected WT mice at 5 days after corneal inoculation. As shown in Fig 4A, the transcription of 117 of 564 immune-related genes were found to be either upregulated or downregulated in whole brain tissues of HSV1-infected *Ripk3*^*-/-*^ mice when compared with whole brain tissues of HSV1-infected WT mice using a minimal threshold of 1.5-fold change or greater. Specifically, transcript abundance was variable in categories related to innate immunity (3 upregulated and 14 downregulated), adaptive immunity (7 upregulated and 8 downregulated), lymphocyte activation (8 upregulated and 11 downregulated), chemokine signaling (5 upregulated and 7 downregulated), cytokine signaling (5 upregulated and 21 downregulated), transcriptional control (3 upregulated and 7 downregulated), oxidative stress (3 upregulated and 0 downregulated), type I and II interferon (IFN) signaling (0 upregulated/ and 5 downregulated) and immunometabolism (3 upregulated and 1 downregulated). Of these, transcripts of cytokines and chemokines known to influence antiviral responses within the CNS were dramatically impacted.

NanoString nCounter analysis findings for select transcripts were confirmed using quantitative real-time RT-PCR (qPCR) assays. Consistent with our NanoString nCounter analysis, IFNγ, which has been shown to be critical for resistance to HSE [40–42], exhibited mRNA levels that were dramatically decreased within whole brains collected from HSV1-infected *Ripk3*^*-/-*^ mice when compared with whole brains collected from HSV1-infected WT mice (Fig 4B) although IFNα/β and tumor necrosis factor (TNF) mRNA levels did not show significant differences (Fig 4A). A qPCR assay analysis of two key chemokines, CXCL10 and CXCL11, also showed reduced levels within whole brains of HSV1-infected *Ripk3*^*-/-*^ mice when compared with levels found within whole brains of HSV1-infected WT mice (Fig 4C). Subsequent protein analysis using ELISA to confirm both NanoString nCounter and qPCR assay mRNA results revealed that IFNγ protein levels were significantly lower in whole brains, brainstems, and blood serum collected from HSV1-infected *Ripk3*^*-/-*^ mice when compared to samples collected from HSV1-infected WT mice (Fig 4D) whereas TNF protein expressions were equivalent for all samples collected from both HSV1-infected *Ripk3*^*-/-*^ mice and HSV1-infected WT mice (Fig 4E).

To further evaluate the contribution of caspase 8 toward production of IFNγ and TNF protein during HSE neuropathogenesis, whole brains, brainstems, and blood serum collected from HSV1-infected *Ripk3*^*K51A/K51A*^*Casp8*^*-/-*^ and HSV1-infected *Ripk3*^*K51A/K51A*^ mice were subjected to analysis by ELISA. Results demonstrated that IFNγ levels were significantly reduced in both brainstem and blood serum samples collected from HSV1-infected *Ripk3*^*K51A/K51A*^*Casp8*^*-/-*^ mice when compared with the same samples collected from HSV1-infected *Ripk3*^*K51A/K51A*^ mice, although no differences were observed in whole brains collected from both animal groups (Fig 4F). TNF protein levels, however, were equivalent for whole brain, brainstem, and blood serum samples collected from both animal groups (Fig 4G). Collectively, these results support the notion that RIPK3, in combination with caspase 8, contributes to a host innate immune response during HSE through the expression of antiviral cytokines and chemokines.

### RIPK3 and caspase 8 together regulate leukocyte infiltration and activation within the brain during HSE

Previous work by others has shown that the chemokine CXCL10 serves an important role in the immunopathogenesis of HSE by driving NK cell as well as T-cell infiltration within the brain parenchyma during HSV1 infection [43]. Because CXCL10 and other important chemokine and cytokine amounts were found to be substantially reduced in mice that showed increased susceptibility to HSE, we next questioned whether a deficiency in RIPK3 or caspase 8 might influence the recruitment of inflammatory cells into the brain during onset of HSE. We therefore used flow cytometric analysis for the detection and quantification of NK cell, CD4+ T-cell, and CD8+ T-cell populations recovered from the whole brains of HSE-susceptible *Ripk3*^*-/-*^ mice and *Ripk3*^*K51A/K51A*^*Casp8*^*-/-*^ mice when compared with HSE-resistant *Ripk3*^*K51A/K51A*^ mice and WT mice, all at 7 days after corneal HSV-1 inoculation.

**Fig 5:**
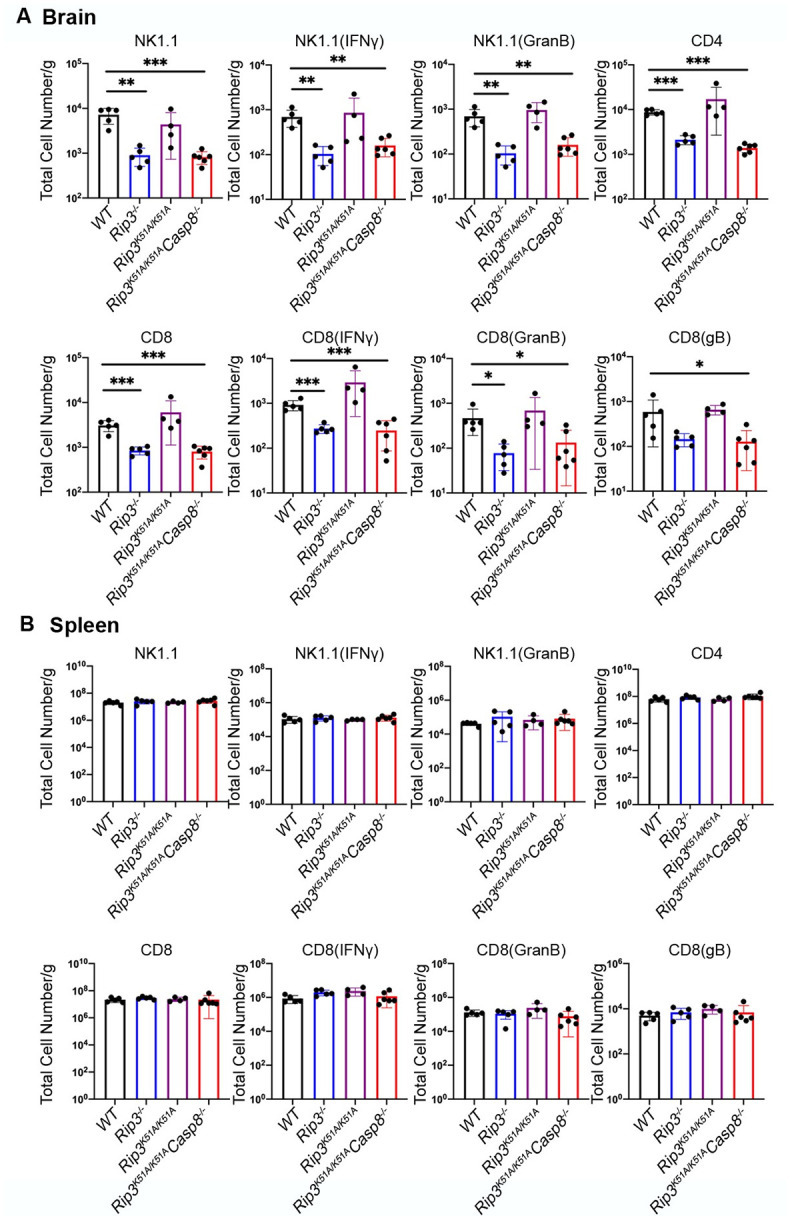
RIPK3 collaborates with Casp8 on NK and T cells recruiting to CNS during HSV1 infection. Numbers of NK, CD4 and CD8 T cells of total brain leukocytes (A) or spleen (B) from WT, *Ripk3*^*-/-*^, *Ripk3* ^*K51A/K51A*^ and *Ripk3*^*K51A/K51A*^*Casp8* ^*-/-*^mice. Total IFNγ^+^ and granzyme B^+^ NK or T cell numbers by intracellular cytokine staining were also evaluated (n = 5–7 per group). All data are presented as mean ± SEM. *P < 0.05; **P < 0.01; ***P < 0.001.

A significant decrease in the recruitment of NK cells was observed in the whole brains of HSE-susceptible HSV1-infected *Ripk3*^*-/-*^ mice and HSV1-infected *Ripk3*^*K51A/K51A*^*Casp8*^*-/-*^ mice when compared with those collected from HSE-resistant HSV1-infected *Ripk3*^*K51A/K51A*^ mice and HSV1-infected WT mice (Fig 5A). The pattern of NK cell recruitment for HSV1-infected *Ripk3*^*K51A/K51A*^ mice was similar to that observed for HSV1-infected WT mice while HSV1-infected *Ripk3*^*K51A/K51A*^*Casp8*^*-/-*^ mice consistently showed a significant reduction in the number of NK cells. Additional flow cytometric analysis directed at NK cell activation markers showed that activation markers IFNγ and granzyme B were significantly reduced along with a proportional decrease in the total number of NK cells detected within the whole brains of HSV1-infected *Ripk3*^*-/-*^ mice as well as HSV1-infected *Ripk3*^*K51A/K51A*^*Casp8*^*-/-*^ mice but not within the whole brains of HSV1-infected *Ripk3*^*K51A/K51A*^ mice and HSV1-infected WT mice (Fig 5A). Similarly, HSE-susceptible *Ripk3*^*-/-*^ mice and *Ripk3*^*K51A/K51A*^*Casp8*^*-/-*^ mice were impaired in their ability to recruit CD4+ T cells and CD8+ T cells into whole brains at 7 days after corneal HSV1 inoculation (Fig 5A). A similar pattern of significantly decreased CD8+ T-cell activation markers assessed by intracellular staining for IFNγ and granzyme B after HSV1 glycoprotein B (gB) stimulation was also observed in the whole brains of HSV1-infected *Ripk3*^*-/-*^ mice and HSV1-infected *Ripk3*^*K51A/K51A*^*Casp8*^*-/-*^ mice (Fig 5A).

Although previous work has shown that *Ripk3*^*-/-*^ mice, *Ripk3*^*K51A/K51A*^ mice, and *Ripk3*^*K51A/K51A*^*Casp8* mice are immunocompetent [36], we nonetheless analyzed whole spleens collected from each of these animal groups for flow cytometric quantification of NK cells, CD4+ T cells, CD8+ T cells and associated activation markers to confirm that systemic immunity was intact in these animals following corneal HSV1 inoculation. Whole spleens recovered from HSV1-infected WT mice served as controls. As expected, no differences in splenic leukocyte populations were observed among the animal groups (Fig 5B). These data indicate that caspase 8 is required to coordinate RIPK3 in the recruitment of antiviral immune cells that include NK cells, CD4+ T cells, and CD8+ T cells into the brain following HSV1 infection during the neuropathogenesis of HSE. This observation is specific to the brain and not observed in spleen during systemic immunity. Taken together, these findings suggest that RIPK3 collaborates caspase 8 to restrict HSE via cell death-independent function correlates with regulation of chemokine and cytokine levels and a subsequent leukocyte recruitment and leukocyte activation to control over higher virus burden in the brains.

## Discussion

Necroptosis is an important mechanism of host control over HSV1 replication. RIPK3 is a well-recognized mediator of TNF-dependent [24], TLR-dependent [27], and virus-induced necroptosis [21], acting as a protein kinase to phosphorylate the effector protein, MLKL [29]. To restrict this PCD pathway in human cells, HSV1 encodes ICP6, a RHIM-signaling modulator that prevents RIPK3-mediated necroptosis during virus infection of human cells, its natural host [22]. In mice, ICP6 acts as a species-dependent inducer of necroptosis by recruiting and activating RIPK3 [44, 45]. ICP6 RHIM-deficient HSV1 does not directly activate RIPK3, but instead induces ZBP1-dependent necroptosis as a sensor of virus RNA in either human cells or mouse cells [46]. HSV1 has been shown to replicate to higher amounts in *Ripk3*^*-/-*^ mouse cells and in *Ripk3*^*-/-*^ mice, thereby implicating RIPK3-mediated necroptosis in host control over infection [37, 44, 45]. Whether RIPK3-mediated necroptosis contributes toward the neuropathogenesis of HSE remains unknown. RIPK3 additionally acts as a scaffold to recruit RIPK1-FADD-Caspase 8 and trigger either apoptosis or death-independent inflammation [30–32]. Our investigation sought to dissect the precise contribution of RIPK3 to HSV1 pathogenesis. In our study, HSV1-infected RIPK3 kinase inactive mice failed to phenocopy RIPK3-deficient mice and remained as resistant to HSE as WT mice. Despite reduced levels of RIPK3, these mice support RIPK3 kinase-independent scaffold function and other consequences of RHIM-dependent signal transduction [30]. The tissue specificity of RIPK3 signaling is also evident in our study. On the one hand, HSV1 replication is restricted by RIPK3 kinase-dependent necroptosis in cornea tissue. On the other hand, *Ripk3*^*K51A/K51A*^*Casp8*^*-/-*^ mice are susceptible to HSE, mirroring the susceptibility of *Ripk3*^*-/-*^ mice and therefore suggesting that RIPK3 scaffold function mediates the recruitment of caspase 8 to dictate virus amounts in the brain during HSE neuropathogenesis.

Although we employed HSV1 as a neurotropic virus in our investigation, our results are reminiscent of findings obtained by others using another neurotropic virus, WNV [33]. This investigation also demonstrated RIPK3 restriction of WNV neuropathogenesis in mice via a cell death-independent mechanism that induced a protective neuroinflammation [33]. Similar to our findings using a mouse model of HSE, enhanced susceptibility to WNV-infected *Ripk3*^*-/-*^ mice was traced to suppressed chemokine production and decreased recruitment of T lymphocytes and inflammatory myeloid cells to the CNS in response to subcutaneous or intracranial virus inoculation but with systemic immunity remaining intact. Importantly, however, our investigation extends these findings to show that RIPK3 scaffold function in dependent upon caspase 8 to restrict HSV1 replication in the brain. Indeed, RIPK3 collaboration with caspase 8 dictates the significant induction of CXCL10 (IP10), a chemokine known to recruit immune cells that restrict HSV1 replication [43, 47]. Mice deficient in CXCL10 (*Cxcl10*^*-/-*^ mice) showed decreased survival following corneal HSV1 inoculation that correlated with elevated amounts of virus within the brain and significant defects in the recruitment of dendritic cells, NK cells, and HSV1-specific CD8+ T cells to the brainstem [42]. This pattern of neuroinflammation using HSV1-infected *Cxcl10*^*-/-*^ mice was consistent with our observation that the brains of HSV1-infected mice deficient in RIPK3 and/or caspase 8 also showed significantly reduced recruitment of NK cells, CD4+ T cells, and CD8+ T cells.

One possibility of RIPK3 scaffold function is that the RIPK3/RIPK1-FADD-Caspase 8 complex drives apoptosis within the brain and restricts HSV1 replication through this PCD pathway. Recently, cGAS/STING-dependent intrinsic apoptosis was implied to operate in microglia and other immune cells during host defense against HSV1 invasion of the brain, most likely through IRF3 recruitment and activation of BAX to initiate intrinsic apoptosis [48, 49]. As an extrinsic apoptosis mediator, activated caspase 8 in some settings is also sufficient to cleave cytosolic Bid. Cleaved Bid (tBid) then translocates to the mitochondria, leading to mitochondria dysfunction and intrinsic apoptosis [50, 51]. Thus, whether caspase 8-mediated extrinsic or intrinsic apoptosis contributes to the restriction of HSV1 replication within the brain requires further clarification.

Another possibility of RIPK3 scaffold function within the CNS is that RIPK3/RIPK1-FADD-Caspase 8 complex-dependent inflammation operates neuroprotection during HSE development. Prior studies have found that HSV1 replicates very well in neurons and astrocytes, but replicates weakly in microglia, cells of the CNS that are mainly responsible for uptake of virus antigen rather than for the support of productive virus replication [9]. Microglial cells as resident macrophages of the CNS are known to secrete pro-inflammatory cytokines and mediate ASC-dependent inflammasome response during HSE development [17, 52]. Thus, it is most likely that virus antigens released from infected neurons or astrocytes activate adjacent microglial cells to stimulate production of chemokines and cytokines that then serve to recruit antiviral NK cells and T cells as well as augment IFNγ production to further control virus infection. While microglia have been studied extensively in the context of the innate immune response to HSV1 infection, recent work also demonstrates that crosstalk between microglia and astrocytes plays an important role in controlling HSV1 replication and neuroinflammation [53]. The relative importance of different CNS cell populations in eliciting RIPK3/RIPK1-FADD-Caspase 8 complex-mediated signaling during HSE neuropathogenesis therefore deserves further investigation.

Although robust expression of RIPK3 has not been detected in the healthy brain [54, 55], RIPK3 has been implicated in the pathogenesis of amyotrophic lateral sclerosis [56] as well as WNV neuropathogenesis in the brain [33]. We therefore hypothesize that HSV1 infection induces RIPK3 expression in the brain during the neuropathogenesis of HSE although its source remains unclear. At least two possibilities present themselves. HSV1 infection could stimulate RIPK3 within one or more neural cell types at time of direct cell infection. Alternatively or simultaneously, infiltrating or circulating hematopoietic cells in response to HSV1 infection could be a source for RIPK3 production. To minimize the risk of compensatory mechanisms, we are presently exploring these possibilities using conditional RIPK3 knockout mice and including in our investigations possible caspase 8 origins as well.

Genetic data from HSE patients have established that the neuropathogenesis of HSE and its possible recurrence can result from as little as a single-gene inborn error of TLR3/IFN pathway-mediated immunity [8]. Our work may expand possible genetic errors to RIPK3 as well as caspase 8 as possible reasons for susceptibility to HSE. Thus, whether the neuropathogenesis of HSE results from single-gene inborn errors of RIPK3 and/or caspase 8 requires further investigation of the possible clinical penetrance of these deficiencies. In summary, we show that HSV1-induced RIPK3-dependent necroptosis appears to operate in the cornea during virus replication. In sharp contrast, virus replication in the brain appears to be controlled by cell death-independent functions of RIPK3 that proceed independently of its pro-necroptotic kinase activity. Moreover, caspase 8 appears to contribute to RIPK3 protection against HSE by regulating chemokine and cytokine production that subsequently mediates recruitment of antiviral leukocyte populations into the HSV1-infected brain parenchyma and provide protection. Our work demonstrates for the first time that the severity of HSE pathology as well as the associated clinical morbidity and mortality are linked to a dysregulation of local immune responses that are governed by RIPK3 scaffold and caspase 8 functions.

## Materials and Methods

### Ethics statement

This study was carried out in adherence to the recommendations in the Guide for the Care and Use of Laboratory Animals of the National Institutes of Health. The protocol was approved by the Institutional Animal Care and Use Committee (IACUC) of Emory University (DAR-2003346-ENTRPR-N).

### Viruses and reagents

The McKrae strain of HSV1 was used throughout this investigation. Virus stocks were prepared in Vero cells and quantified in Vero cells as described previously [15]. Vero cells were grown and maintained in Dulbecco’s Modified Eagle Medium (DMEM) supplemented with 100 IU/ml penicillin and100 μg/ml streptomycin. Quantification of infectious virus was accomplished by seeding Vero cells into 12-well plates at a density of 3×10^5^ at 24 hrs prior to virus inoculation. After reaching ~90 to 100% confluency, cells were inoculated with 10-fold serial dilutions of virus, incubated for 1 hr at 37°C with the rocking of plates every 15 min to ensure even distribution of virus, followed by the addition of 2 ml of medium containing 2% methycellulose. Cell monolayers were stained with crystal violet, and plaques were counted at 72 hrs after inoculation.

### Mice

*Ripk3*^−/−^ [57], *Ripk3*^*K51A/K51A*^ [30], *Ripk3*^*-/-*^ *Casp8*^*−/−*^, and *Ripk3*^*K51A/K51A*^ *Casp8*^*−/−*^ [30] mice (knockout mice) have been described previously and backcrossed to >98% on the C57BL/6J background. C57BL/6J WT mice were purchased from Jackson Laboratories and bred at Emory University Division of Animal Resources. Age-matched mixed groups of 6 to 8-week-old female and male mice were used during the performance of individual experiments.

### Mouse infections and survival studies

The left eyes of groups of adult (6 to 8 weeks old) knockout mice and WT mice of both sexes were anesthetized, scarified with a 25-gauge needle, and inoculated with 5 ul of either 5 X 10^5^ plaque-forming units (PFU) of HSV1 [McKrae] contained within DMEM or mock-infected with 5 μl of DMEM only as described previously [17]. Following corneal inoculation, previous studies by others have shown that virus travels by neural routes to the adjacent trigeminal ganglion and from there into the cerebrum and cerebellum of the brain via the brainstem [58]. For survival studies, groups of HSV1-infected mice were observed daily for up to 20 days after corneal inoculation for death or for the appearance of clinical signs and symptoms of acute encephalitis (hunched posture, moribund appearance, and/or seizures) and euthanized. This study was carried out in adherence to the recommendations in the Guide for the Care and Use of Laboratory Animals of the National Institutes of Health. The protocol was approved by the Institutional Animal Care and Use Committee (IACUC) of Emory University (DAR-2003346-ENTRPR-N).

### Virus quantification of tear films and neurologic tissues

Tear films, trigeminal ganglia, the entire brainstem, and whole brain (cerebrum and cerebellum) from individual animals of different animal groups were assessed for amounts of infectious virus at 5 days after corneal HSV1 inoculation. Tear films were collected by gently pressing the eye and wiping a sterile cotton swab three times around the eye in a circular motion and twice across the center of the corneal surface. The swabs were then placed in 1 ml of DMEM, subjected to quantitative plaque assay, and reported as PFU/ml/swab. Collected brain tissues were individually placed in 1 ml of DMEM, individually homogenized, subjected to quantitative plaque assay, and reported as PFU/ml/brain tissue.

### Histopathology

Entire brainstems were collected from different animal groups at 7 days after corneal HSV1 inoculation and analyzed and compared histopathologically. Briefly, mice were perfused transcardially with cold phosphate-buffered saline and post-fixed with phosphate-buffered 4% formaldehyde. Entire brainstem tissues were removed, paraffin-embedded, sectioned, and stained with hematoxylin and eosin (H&E) at the Histology and Molecular Pathology Laboratory of Emory University Yerkes National Primate Research Center. H&E-stained brainstem sections from different animal groups were then scored in a blinded fashion by a pathologist using a scoring system (0–4) with increasing severity developed by us for quantification of HSE-associated pathologic markers that included the appearance of microglial nodules (gliosis), perivascular cuffing, and degree of meningeal inflammation.

### NanoString gene expression analysis

NanoString nCounter gene expression analysis was performed as described by us previously [59]. In brief, total RNA was isolated from a 2-mm-thick brain section taken from the left hemisphere of the cerebrum using an RNA Mini Kit (Invitrogen). One hundred ng of total RNA were then analyzed using the nCounter Analysis System (NanoString Technologies, Seattle, WA) according to the manufacturer’s instructions in combination with the Murine Immunology Panel which contained 564 unique RNA barcodes. Probes for 15 internal and housekeeping genes such as ribosomal protein L10, beta-actin, beta-2-microglobuin, glyceraldehyde 3-phosphate dehydrogenase, and ribosomal protein L19 were incorporated into the NanoString codesets of this panel. Analysis of raw mRNA data was performed using the NanoString nSolver^™^ analysis software version 4.0. Fold changes greater than 1.5 or *p* ≤ 0.05 were included for analysis. A heat map was generated with grouped values determined by using the mean of the gene expression levels. For heat map representation, the expression level of each gene was log^2^ which was then z-score-transformed using a custom R script.

### Quantitative real-time RT-PCR assay

Total RNA was isolated from homogenates of whole brains collected from different animal groups at 5 days after corneal HSV1 inoculation using Qiagen’s Rneasy Mini Kit (74104). Following reverse transcription to yield cDNA using PrimeScript RT reagent kit (RR037A, Takara), samples were amplified using PrimeTime Gene Expression Master Mix (1055772, IDT) on a CFX Connect Real-Time PCR detection system (1855201, BioRad). Primer sets of IFNα, INFβ, INFγ, TNF, CXCl10, CXCL11, and glyceraldhyde 3-phosphate dehydrogenase (GAPDH) for detection and quantification of transcript were purchased from IDT. The parameters for quantitative real-time RT-PCR assay cycles were 10 min at 95°C, followed by 35 cycles at 15 sec at 94°C, 31 sec at 55°C, and 35 sec at 70°C. Data analysis of genes was performed using the comparative ΔΔCt method (relative mRNA expression, or fold change), with GAPDH acting as the endogenous housekeeping gene for each sample. Samples from mock-infected animal groups were used as control comparison groups.

### ELISA

Whole brain (cerebrum and cerebellum) and entire brainstem collected from different animal groups at 5 days after corneal HSV1 inoculation were homogenized and clarified by centrifugation. Fifty μl of the resulting supernatant as well as serum samples collected from the same animal groups were quantified and compared for TNFα and IFNγ protein levels using a commercially available ELISA kit (R&D, DY410 for TNFα and DY485 for IFNγ) according to the manufacturer’s instructions after analysis at 405 nm using a BioTek Synergy HT microplate reader. Data analysis was performed using associated Gen5 data analysis software.

### Flow Cytometry

At 7 days after corneal HSV1 inoculation, mice from different animal groups were euthanized and perfused transcardially using cold phosphate-buffered saline until the flow-through was clear for removal of intravascular leukocytes. For isolation of leukocytes from brain tissues, whole brains were removed, cut into small pieces, incubated in Hank’s balanced salt solution (HBSS) containing 25 μg/ml of Liberase (Roche) and 10 μg/ml of DNase I (Worthington) for 30 minutes at 37°C, filtered through a 100 μm pore filter, suspended in 10 ml of 30% Percoll (GE Healthcare) in Roswell Park Memorial Institute (RPMI) 1640 medium which was gradually overlaid on top of 2 ml of 70% Percoll, and centrifuged at 500g for 30 minutes at room temperature. The resulting myelin debris was removed from the top of the gradient and discarded; the resulting layer of leukocytes was collected and washed. The total number of viable leukocytes was determined by the trypan blue exclusion test. Single-cell suspensions were also prepared from spleens collected from the same animal groups as previously described [60]. For evaluation of antiviral cytokine production, cells were incubated at 37 °C for 5 hr with 10^−6^ M HSV1 glycoprotein B (gB) (SSIEFARL) peptide (JPT Technologies) in the presence of GolgiStop (BD Biosciences) before staining. Antibodies used were CD8-FITC (553030, 1:100), IFNγ-FITC (554411, 1:125), TNF-PECy7 (557644, 1:100), CD8 PerCPCy5.5 (551162, 1:100), NK1.1-PE-Cy7 (552878, 1:80), CD11b-APC-Cy7 (557657, 1:100), CD3-Pacifc Blue (558214, 1:125), CD19-FITC (557398, 1:100), and B220-PerCp (553093, 1:100) purchased from BD Biosciences; CD3-PECy7 (25-0032-82, 1:125), B220-APC (17-0452-82, 1:100) purchased from eBioscience; CD8-APC (100712, 1:100), and CD4-PE (100408, 1:125) purchased from BioLegend; and CD45-PE-TexasRed (MCD4517, 1:200) purchased from Invitrogen. Data were acquired using an LSRII flow cytometer (BD Biosciences) and analyzed with FlowJo software.

### Statistics

All statistics were calculated using GraphPad Prism 7.0 software. For survival experiments, log-rank analysis was performed. Viral titers and cytokines were analyzed using unpaired two-tailed t-tests for comparisons. Gene expression array results were analyzed using the delta-delta Ct method with significant results determined by t-tests of 2^-ΔCt^ values for each gene. Flow cytometry results were analyzed using one-way ANOVA with Dunnett’s multiple comparison test.
